# Workplace interventions focusing on how to plan, organize and design the work environment in hospital settings: A systematic review

**DOI:** 10.3233/WOR-230205

**Published:** 2024-06-07

**Authors:** Patrik Haraldsson, Elisabeth Nylander, Dirk Jonker, Axel Ros, Kristina Areskoug Josefsson

**Affiliations:** a Occupational Safety and Health Care, Region Jönköping County, Jönköping, Sweden; b School of Health and Welfare, Jönköping Academy for Improvement of Health and Welfare, Jönköping University, Jönköping, Sweden; c Jönköping University Library, Jönköping University, Jönköping, Sweden; d Futurum –Academy for Healthcare, Region Jönköping County, Jönköping, Sweden; e University West, Department of Health Sciences, Trollhättan, Sweden; f Department of Behavioural Science, Oslo Metropolitan University, Oslo, Norway

**Keywords:** Health personnel, occupational health services, occupational health, health promotion, implementation science, working conditions

## Abstract

**BACKGROUND:**

Occupational Health Service (OHS) is a service that should support employers and employees with their work environment. Previous research indicates the need for deeper knowledge about the effect of workplace interventions with a focus on planning, organizing and designing the workplace to improve work conditions in hospital settings.

**OBJECTIVE:**

The aim was to evaluate the outcomes, workplace interventions and intervention strategies in hospital settings.

**METHODS:**

A systematic literature review was conducted. CINAHL, MEDLINE, PsycInfo, Scopus, and Web of Science Core Collection were searched in September 2021. The Mixed Methods Appraisal Tool was used to evaluate the quality of the included studies. Study results are presented through a narrative synthesis. A protocol for this study was registered on the Open Science Framework.

**RESULTS:**

Twenty-six studies, published between 2010 and 2021, were included. These included randomized controlled trials (RCTs), non-RCTs, and mixed methods reports with moderate to good quality. The results support the use of workplace interventions to improve work conditions, health, and well-being in hospital settings. Combinations of different interventions, tailored to the specific organization, were used. Important intervention strategies commonly used in the start-up, evaluation, and intervention of successful workplace interventions, were identified. Using a pragmatist complexity approach in workplace interventions can improve outcomes by providing clear intervention strategies and combinations of tailored interventions, related to context specific problems.

**CONCLUSION:**

OHS support in workplace interventions with clear intervention strategies will contribute to improve work conditions, health and well-being in hospital settings.

## 1 Introduction

The United Nations Sustainable Development Goals (UN-SDG) 8.8 is about promoting safe and secure working environments for all employees [1]. The work environment is a complex phenomenon consisting of many factors which affect employees. Considerations must be given to physical, psychological and psychosocial factors to understand risk exposure [2]. These aspects of the work environment are intertwined and affect employees in different ways [3, 4]. This is exemplified by the strong associations between workload, job control, decision authority, social support at work, and chronic low back pain [4], or by showing that both intensity and frequency exposure of heavy lifting were associated with low back pain [3].

Occupational Health Service (OHS) is a service that should support employers and employees with their work environment. According to International Labour Organization (ILO), OHS is a service with essentially preventive functions to support work to establish and maintain healthy working environment and adapting work to the employees [5]. However, there is a gap between the ILO definition and OHS work in practice. In general, the OHS does not work in accordance with the ILO definitions, as shown in international research [6, 7]. OHS contribution to promote good working conditions is limited and this stresses the need of further development and improvement of OHS services [6, 7]. According to ILO important functions of OHS are: surveillance and identification of risk factors in the workplace which may affect the employees’ health, advice on planning and organization of work, including the design of workplaces, and promoting the adaption of work to the employees [5]. Due to the complex and intertwined nature of work environments a pragmatist complexity approach was used it this study. It suggests that problem-solving must be more attentive to context when problems become more complex [8]. In a pragmatist complexity approach, both adequate interventions and adequate intervention implementation strategies must be identified to successfully transfer knowledge useful for OHS practice.

The definition for *workplace interventions* used in this study is interventions with a focus on planning, organizing and designing the workplace to improve work conditions, as described by ILO [5]. This is a novel approach compared to studies which focus on interventions for individuals, such as exercise or stress reduction training.

The work environment in hospitals has been highlighted during the COVID-19 pandemic, but high demands at work is not a new phenomenon in hospitals [9]. In hospital settings, the work environment has been shown to affect employees in the form of a high workload, long work shifts, low sense of control [10], and lifting, pushing and pulling during patient handling [11]. Exposures like these might negatively affect employee health [3, 10]. This shows a gap between the ambitions in UN-SDG 8.8 and work environment reality in hospitals.

There is evidence to support workplace interventions (reducing workload, enhancing teamwork and leadership, changing work schedules, clinical supervision) to reduce stress, distress and burnout in physicians and nurses in hospital settings [12–16]. There is some evidence to support a participatory approach to improve nurse work conditions, such as tailoring interventions to the organization and implementing iterative processes as intervention strategies [17]. Otherwise, there is a lack of evidence to benefit the work environment in hospital settings.

The results show knowledge gaps with regards to the work environment in hospital settings in a broader sense. There is a need for more knowledge about physical outcomes, psychosocial outcomes (including other than stress, distress and burnout). There is a need for knowledge with regards to hospital workers in general and not only physicians or nurses. There is also a need for a deeper understanding of how workplace interventions and intervention strategies affect work conditions and work-related health outcomes.

Increased knowledge about employee and work-related outcomes, workplace interventions and intervention strategies in hospital settings are needed to improve the ability of OHS to work in accordance with ILO definitions. Improvement in OHS work will support efforts according to the UN-SDG 8.8 by creating safer workplaces to benefit employers and employees in hospital settings.

### 1.1 Aim

The aim of the study was to summarize the evidence for workplace interventions with regards to outcomes, workplace interventions and intervention strategies in hospital settings.

Research question 1: How do workplace interventions affect employee and work-related outcomes in hospital settings?

Research question 2: Which workplace interventions and intervention strategies are used in workplace interventions in hospital settings?

## 2 Methods

### 2.1 Study design

A systematic literature review with a narrative summary [18]. This study is presented according to the Preferred Reporting Items for Systematic reviews and Meta-Analyses (PRISMA) 2020 statement [19].

### 2.2 Eligibility criteria

Studies published in English between January 2010 and September 2021 were considered. Research indicates a long-term gradual increase of complexity in hospital settings [20, 21]. The start date of the searches was a pragmatic choice used to increase the relevance of the included studies. The search strategy was structured according to PICO (population, intervention, comparators, and outcome).

The population in the included studies were employees of all professions working within hospitals (e.g., physicians, nurses, nursing assistants, medical administrators, occupational therapists, physiotherapists, technicians). The intervention were workplace interventions (randomized controlled trials (RCTs), non-RCTs and mixed methods studies) focusing on planning, organizing and designing the workplace context to benefit work conditions and employee health. Intervention studies with any comparator/no comparator were included. To be included, studies where work conditions and work-related outcomes (e.g., stress, burnout, physical pain, or sick leave) were evaluated.

Inclusion criteria: Workplace interventions (RCTs, non-RCTs and mixed methods) focused on planning, organizing and designing the workplace context aiming to improve work conditions and employee health in hospital settings were included. Exclusion criteria: Individual focused work environment interventions intended to support the hospital employee, such as coping strategies to manage work demands, were excluded. Study designs other than intervention studies were excluded.

### 2.3 Information sources and search strategy

Literature search strategies were developed and conducted by a research librarian (EN) to reflect the concepts outlined. A combination of title and abstract keywords were used as well as controlled vocabulary whenever possible. A set of key articles were identified before the search process and were used to generate search terms and to test the effectiveness of the strategies in each database. The MEDLINE strategy was developed with input from the project team, then peer reviewed by a second librarian, not otherwise associated with the project. After the MEDLINE strategy was finalized, it was then adapted to the syntax and subject headings of the other databases/platforms.

The following databases/platforms were searched in September 2021: CINAHL with Full Text (EBSCOHost), MEDLINE (EBSCOHost), PsycInfo (ProQuest), Scopus (Elsevier), and Web of Science Core Collection (SCI-EXPANDED, SSCI, A&HCI, CPCI-S, CPCI-SSH, ESCI). See appendix for complete documentation of the search strategies. In addition, the CENTRAL trials registry of the Cochrane Collaboration (Wiley) was searched for ongoing or recently completed trials. Before undertaking this review and to avoid research waste, the PROSPERO database and Google Scholar were also searched for ongoing or recently completed systematic reviews on the same topic. The searches were limited to intervention studies published in English from 2010 onwards. As relevant studies were identified, the reviewers checked for additional relevant cited and citing articles.

### 2.4 Selection process

Records found during the search phase were exported to a reference management software (EndNote) to enable the identification and removal of duplicates [22]. Prior to the formal screening process, a calibration exercise was undertaken to pilot and refine the screening questions. Records were then screened using Rayyan, a web-based application for systematic reviews [23] based on the previously described inclusion/exclusion criteria. The primary investigator (PH) performed an initial screening on the title/abstract level. The last author (KAJ) screened random samples, as well as titles/abstracts with uncertainties. Disagreements were resolved by discussion until consensus was reached. Two team members (PH and KAJ) independently conducted full-text assessment of included records, and any disagreements were resolved by discussion until consensus was reached.

### 2.5 Quality assessment

Full-text studies were analyzed independently by two authors (PH and KAJ) with the Mixed Methods Appraisal Tool (MMAT). MMAT is a critical analysis tool for studies, allowing studies with different study designs to be included in a systematic literature review. It permits to appraise the methodological quality of five categories of studies: qualitative research, randomized controlled trials, non-randomized studies, quantitative descriptive studies, and mixed methods studies [24].

The quality assessment with MMAT was conducted in two steps. The first step was conducted by responding to two screening questions similar for all study designs (S1. Are there clear research questions? S2. Do the collected data allow to address the research questions?). The response options were *yes*, *no* or *can't tell*. Responding no or can't tell to one or both screening questions might indicate that the paper is not an empirical study, and thus cannot be appraised using the MMAT. The second step was conducted by choosing the correct study category (RCT, non-RCT or Mixed methods in our study). Regardless of study design each study was evaluated by five quality criteria questions, with the response options yes, no or can't tell. The can’t tell response category meant that the paper did not report appropriate information to answer yes or no [24]. Any disagreements during the analysis process were resolved by discussion until consensus was reached.

In the MMAT it is discouraged to calculate an overall score from the ratings of each criterion. Instead, it is advised to provide a more detailed presentation of the ratings of each criterion to better inform the quality of the included studies [24].

### 2.6 Data extraction

Data on outcome and interventions were extracted from the intervention studies. Data on intervention strategies were extracted with a matrix of intervention strategies based on principles from Gustafson and von Thiele Schwarz [25, 26]. Start-up criteria, evaluation methods, interventions, and outcome characteristics were evaluated in each study. The matrix contained twenty-six items answered with yes, no/unknown or not applicable. Data extraction was conducted independently by two authors (PH and DJ). Any disagreements during data extraction were resolved by discussion until consensus was reached.

### 2.7 Data synthesis

Since a large heterogeneity in outcomes and interventions were expected, the data synthesis was conducted with a narrative summary [18]. Employee outcomes were presented in three categories: physical outcomes, psychosocial outcomes and other outcomes, together with related workplace interventions. Intervention strategies were presented in three categories: start-ups, evaluation methods and intervention characteristics.

## 3 Results

### 3.1 Study selection

**Fig. 1:**
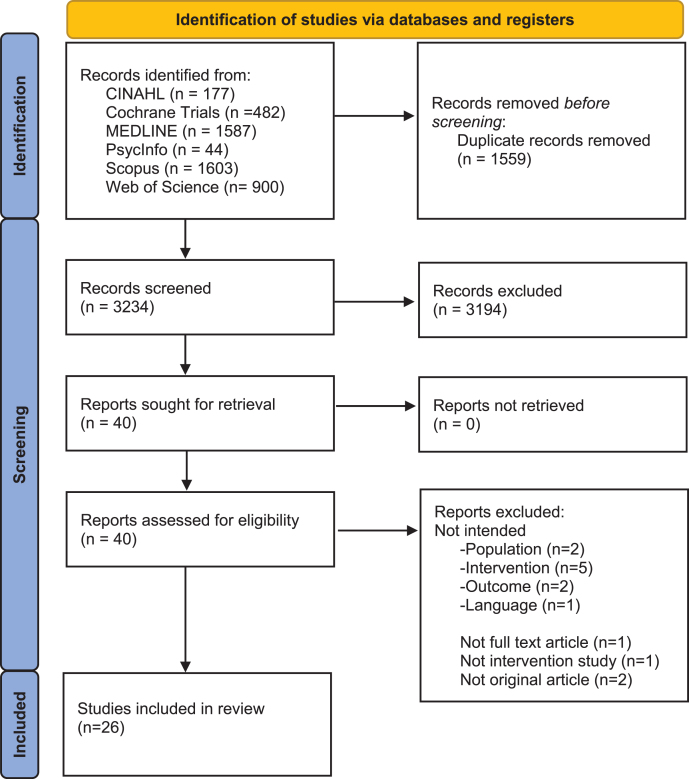
PRISMA 2020 flow diagram of review process.

The database searches identified 4,793 records. After the removal of duplicates, 3,234 records were screened on the title/abstract level. Of these, 40 full-text documents were reviewed, and finally 26 papers [27–52] were included for analysis. See Fig. 1 for a flow diagram of the review process.

### 3.2 Characteristics of the included studies

The study design of the included studies were RCTs (*n* = 12), non-RCTs (*n* = 13), and mixed methods (*n* = 1), published between 2010 and 2021 with a study duration between 4 weeks and 13 years. The studies were conducted in Europe (Italy, France, The Netherlands, Denmark, and Sweden), Asia (Thailand, China, Korea, Vietnam, and Japan), Canada and the USA. The sample sizes ranged from *n* = 8 to *n* = 11545 (Table 4).

### 3.3 Quality assessment

The quality assessment showed that all the included RCTs, except Bagaipour-Divshali [28], had an appropriately performed randomization. The RCT by West [51] did not have comparable groups at baseline and the study by Sohn [47] did not present data on baseline characteristics. Complete outcome data were presented in all the included RCTs. The quality assessment identified a general lack of blinding in the included RCTs. Only five of twelve RCTs clearly stated that the assessors were blinded to the intervention. The study by van der Meer [49] did not have blinded assessors and no information about assessor blinding was presented by Bagaipour-Divshali [28], Chanchai [31], MacIntyre [40], Uchiyama [48], van Der Molen [50] and West [51]. All included RCTs, but Bagaipour-Divshali [28] and Chanchai [31], presented data showing that the participants adhered to the assigned intervention (Table 1).

**Table 1:** Evaluation of study quality in quantitative randomized controlled trials (RCTs), with Mixed Methods Appraisal Tool (MMAT). Specific items are addressing study quality of the RCTs. All items in MMAT are answered yes, no or can’t tell

Author (year) Items	Bagaipour-Divshali (2016)	Chanchai (2016)	El Khamali (2018)	Jacobsen (2019)	Lee (2013)	Macintyre (2014)	Macintyre (2015)	Sohn (2018)	Uchiyama (2013)	Van der Meer (2015)	Van Der Molen (2011)	West (2014)
2.1. Is randomization appropriately performed?	No	Yes	Yes	Yes	Yes	Yes	Yes	Yes	Yes	Yes	Yes	Yes
2.2. Are the groups comparable at baseline?	Yes	Yes	Yes	Yes	Yes	Yes	Yes	Can't tell	Yes	Yes	Yes	No
2.3. Are there complete outcome data?	Yes	Yes	Yes	Yes	Yes	Yes	Yes	Yes	Yes	Yes	Yes	Yes
2.4. Are outcome assessors blinded to the intervention provided?	Can't tell	Can't tell	Yes	Yes	Yes	Can't tell	Yes	Yes	Can't tell	No	Can't tell	Can't tell
2.5 Did the participants adhere to the assigned intervention?	Can't tell	Can't tell	Yes	Yes	Yes	Yes	Yes	Yes	Yes	Yes	Yes	Yes

The quality assessment showed that all non-RCTs had participants that were representative of the target population and that the measurements were appropriate in relation to outcome and intervention. All the included non-RCTs, except d’Ettore [32], presented complete outcome data. Confounders were accounted for in all studies, except Pierce [45]. In Mehrdad [42] the intervention was not administered as intended and Abdollahi [27] and d’Ettore [32] did not present information about this. All other non-RCTs administered the interventions as intended (Table 2).

**Table 2:** Evaluation of study quality in non-randomized trials (non-RCTs), with Mixed Methods Appraisal Tool (MMAT). Specific items are addressing study quality of the non-RCTs. All items in MMAT are answered yes, no or can’t tell

Author (year) Items	Abdollahi (2020)	Black (2011)	Bourbonnais (2011)	dÉttore (2016)	Gordon (2018)	Graeve (2017)	Guay (2016)	Jia (2020)	Mehrdad (2013)	Niks (2018)	Pierce (2021)	Schoenfisch (2013)	Zadvinskis (2010)
3.1. Are the participant’s representative of the target population?	Yes	Yes	Yes	Yes	Yes	Yes	Yes	Yes	Yes	Yes	Yes	Yes	Yes
3.2. Are measurements appropriate regarding both the outcome and intervention (or exposure)?	Yes	Yes	Yes	Yes	Yes	Yes	Yes	Yes	Yes	Yes	Yes	Yes	Yes
3.3. Are there complete outcome data?	Yes	Yes	Yes	Can't tell	Yes	Yes	Yes	Yes	Yes	Yes	Yes	Yes	Yes
3.4. Are the confounders accounted for in the design and analysis?	Yes	Yes	Yes	Yes	Yes	Yes	Yes	Yes	Yes	Yes	No	Yes	Yes
3.5. During the study period, is the intervention administered (or exposure occurred) as intended?	Can't tell	Yes	Yes	Can't tell	Yes	Yes	Yes	Yes	No	Yes	Yes	Yes	Yes

One study with mixed methods design, by Michélsen [43] was included. The quality assessment showed that the study had an adequate rational for the use of mixed methods design to address the research question. The quality assessment also showed that the different components of the study were effectively integrated to answer the research question. The study did not present data to evaluate if the integration of qualitative and quantitative components were adequately interpreted. The divergences and inconsistencies between qualitative and quantitative data were not adequately addressed. The study did adhere to quality criteria in the qualitative and quantitative traditions (Table 3).

**Table 3:** Evaluation of study quality in mixed methods trials, with Mixed Methods Appraisal Tool (MMAT). Specific items are addressing study quality of the mixed methods trial. All items in MMAT are answered yes, no or can’t tell

Author (year) Items	Michélsen (2014)
5.1. Is there an adequate rationale for using a mixed methods design to address the research question?	Yes
5.2. Are the different components of the study effectively integrated to answer the research question?	Yes
5.3. Are the outputs of the integration of qualitative and quantitative components adequately interpreted?	Can't tell
5.4. Are divergences and inconsistencies between quantitative and qualitative results adequately addressed?	No
5.5. Do the different components of the study adhere to the quality criteria of each tradition of the methods involved?	Yes

### 3.4 Workplace interventions and outcomes

Improvements were identified in a wide variety of outcomes. Statistically significant improvements were found in **physical outcomes** like decreases in pain, discomfort, musculoskeletal disorders, and injuries [27, 29, 31, 39, 46, 47, 52], as well as increases in the use of patient handling equipment [37, 52]. The interventions were heterogeneous and included ergonomic education [29], ergonomic education/practical ergonomic changes [27, 31], work stress reduction [27], workstation re-design [31], changes in administrative work [31], minimal lift policy and lift equipment [46, 52] and minimal lift support (peer-coaches) [52]. The Jacobsen study was an exception concerning positive outcomes, as they did not find any changes in low back pain or injuries despite improvement in the use of assistive devices [37]. Interventions related to physical outcomes consisted of different combinations of these interventions (Table 4).

**Table 4:** Study characteristics, workplace interventions and outcomes in the individual studies

Study ID		Population		Study result	
First author (year)	Study design (Duration)	Country	Sample size	Workplace interventions^1^	Outcomes^2^
Abdollahi (2020)	Non-RCT (3 months)	Iran	*n* = 74	Ergonomics educational program. Empowering employees in participating to improve ergonomics and work stress.	Decreased pain in neck, shoulder, low back etc. Decreased risk of MSD.
Babaeipour-Divshali (2016)	RCT (3 months)	Iran	*n* = 60	Head nurse empowerment program. Improving management knowledge and skills.	Increased nurse job satisfaction.
Black (2011)	Non-RCT	Canada	*n* = 2809	Patient handling equipment and education aimed to maximize the use of the equipment.	Decreased injury rates. Decreased time loss at work due to injuries.
Bourbonnais (2010)	Non-RCT (3 years)	Canada	*n* = 1568	Participatory intervention teams of hospital employees. Identifying and improving psychosocial factors at work (task rotations, bed reductions, ergonomics, communications etc.).	Improvement in psychosocial work factors (psychological demands, supervisor support, work related burnout etc.).
Chanchai (2016)	RCT (5 months)	Thailand	*n* = 100	Ergonomics educational program. Empowering employees in participating to improve ergonomics, workstation design, and administration.	Decreased prevalence of MSDs (upper- and lower back). Improvements in work pace, meaning of work, support form supervisor etc.
dÉttorre (2016)	Non-RCT (3 years)	Italy	*n* = 765	Organizational interventions focused on team reduce work related stress.	Decreased number of needle stick injuries. Cost savings from managing fewer needle stick injuries.
El Khamali (2018)	RCT (6 months)	France	*n* = 198	Multimodal program including education, role-play and debriefing to reduce job stress.	Decreases in job strain, isostrain (job strain/low social support) and absenteeism.
Gordon (2018)	Non-RCT	The Netherlands	*n* = 177	Workshops in job crafting (seeking challenges, seeking resources and reducing demands) improving teamwork.	Increases in work engagement and health. Decreases in exhaustion.
Graeve (2017)	Non-RCT (10 months)	USA	*n* = 163	Policy update and participatory intervention (PDSA) to improve antineoplastic drug safe handling.	A decrease of contaminated surfaces after interventions
Guay (2016)	Non-RCT (10 months)	Canada	*n* = 89	The Omega program. Education aimed at empowering hospital workers to minimize patient aggression directed toward hospital workers.	Decreases in exposure to minor violence, exposure to violent acts and psychological distress.
Jacobsen (2019)	RCT (12 months)	Denmark	*n* = 625	Participatory intervention to implement department specific solutions (insufficient time, outdated equipment, and lack of space etc.) for improving the use of assistive devices in patient transfers.	Increases in general use of assistive devices. Increases in collegial encouragement, discussions and guidance about the use of assistive devices in patient transfers. No difference in use of necessary assistive device, low back pain and back injuries.
Jia (2020)	Non-RCT (21 months)	China	*n* = 435	Restricted access policy to decrease violence towards hospital workers.	Decreases in psychological violence towards hospital workers.
Lee (2013)	RCT	Korea	*n* = 8	Evaluating different operating table heights on the quality of laryngeal view and discomfort in anesthetists.	Higher operating tables decreased the intubation discomfort in anesthetists. Lower operating tables decreased the mask ventilation discomfort in anesthetists.
MacIntyre (2014)	RCT (4 weeks)	China	*n* = 1922	Comparing N95 respirators and medical masks to each other and to controls who did not routinely wear masks.	N95 respirators were protective against bacterial colonization, co-colonization, viral-bacterial co-infection and dual virus infection.
MacIntyre (2015)	RCT (4 weeks)	Vietnam	*n* = 1607	Comparing cloth masks to medical masks and controls without masks.	The rates of laboratory confirmed virus infections were lowest in the medical masks, followed by the control arm, and highest in the cloth masks.
Mehrdad (2013)	Non-RCT (3 years)	Iran	*n* = 1456	Training course on how to prevent needle stick injuries in hospital A. Hospital B acted as a control.	Increases of needle stick injuries in hospital A.
Michélsen (2014)	Mixed methods (3.5 years)	Sweden	*n* = 373	Management and employee participation in identifying mental strains at work and support tailored interventions (divisions of work duties, cooperation, personal development at work etc.)	Decreases of mental strain in two groups (doctors and midwives). Decreases in opportunity to influence decisions. Worsened social climate among nurses.
Niks (2018)	Non-RCT	The Netherlands	*n* = 111	Participatory intervention with the DISCovery method in three steps. 1. Identifying psychosocial risks. 2. Develop interventions. 3. Implement the interventions (lean, work breaks, job crafting, cooperation/communication, supervision etc.).	Improvement in work related aspects like emotional- and physical resources, work-break conditions, teamwork, work satisfaction etc.
Pierce (2021)	Non-RCT (12 months)	USA	*n* = 262	Employee participation in identifying work aspects and re-design the work environment to reduce burnout and enhance well-being (leadership, workflow processes, use of technology, team culture etc.).	Decreases in emotional exhaustion. Increases in recommending the workplace as a good place to work.
Schoenfisch (2013)	Non-RCT (13 years)	USA	*n* = 11545	Implementation of mechanical lift equipment and a minimal manual lift policy.	Decreases in injury rate at the community hospital. No change in injury rate at the medical center.
Sohn (2018)	RCT	Korea	?	Evaluation of optimal height of the operating table in anesthesiologists at spinal anesthesia.	Higher operating tables reduces discomfort and joint flexion in anesthesiologists.
Uchiyama (2013)	RCT (6 months)	Japan	*n* = 434	Employee participation in identifying good work examples and implementing them to improve the psychosocial work environment (clarified goals and responsibilities, reward good work, support poor work etc.).	Improvements in co-worker support and realistic goals. No change in mental health status.
Van der Meer (2015)	RCT (12 months)	The Netherlands	*n* = 1649	Education and participatory working groups to prevent hand eczema.	The intervention group were more likely to report hand eczema and showed a more preventive behavior.
Van der Molen (2011)	RCT (12 months)	The Netherlands	*n* = 529	Evaluation of needle safety device and workshop (education), workshop only and control to prevent needle stick injuries.	Decreases in self-reported needle stick injuries and improved safety culture between intervention groups and control.
West (2014)	RCT (12 months)	USA	*n* = 74	Small discussion/reflection groups to improve well-being at work.	Increases in engagement at work and decreases in depersonalization.
Zadvinskis (2010)	Non-RCT (12 months)	USA	*n* = 161	Minimal lift equipment, policies and support (peer-coaches) to decrease patient handling injuries.	Increases in use of patient handling equipment. Decreases in patient handling injuries.

^1^Workplace interventions focused on planning, organizing and designing the workplace context (in accordance with PICO). ^2^Outcomes, regarding work conditions and work-related problems such as stress, burnout, physical pain or sick leave (in accordance with PICO).

Two studies of ergonomic interventions evaluating operating table height for anesthetists, were exceptions as they did not consist of combination interventions [39, 47]. Higher operating tables reduced discomfort [47], and decreased intubation discomfort [39] and lower operating tables decreased ventilation discomfort [39] (Table 4).

Statistically significant improvements were found in **psychosocial outcomes** like decreases in work-related burnout and improvements in psychosocial work factors like demands, support, and job satisfaction [28, 30, 33, 34, 43–45, 48, 51]. The interventions were heterogeneous and included organizational interventions like job crafting [34, 44], task rotations [30], bed reductions [30], lean improvement [44], work breaks [44], improvement of workflow processes [45], and divisions of duties [43]. Psychosocial interventions like leadership [28, 45], cooperation/communication [30, 43], team culture [34, 45], rewards [48], support [48], role play [33], debriefing [33], reflection groups [51], and clarifications of responsibilities and goals [48] were also used. All interventions related to psychosocial outcomes consisted of different combinations of these interventions (Table 4).

Statistically significant improvements were also found in **other outcomes**. Education to reduce needle stick injuries, as a single intervention, increased the number of reported needle stick injuries [42]. A combination of education and needle stick devices decreased the number of needle stick injuries more than only education [50]. Interventions to reduce work stress decreased the number of needle stick injuries further [32] (Table 4).

Exposure to work violence decreased through education in an intensive and emergency department [36] and through a restricted access policy in a tertiary hospital [38]. Education and participative working groups to prevent hand eczema led to a more preventive behavior [49]. A policy update in combination with employee participation in improving drug handling decreased the number of contaminated surfaces [35] (Table 4).

Work-related viral/bacterial infections decreased with the use of N95 respirators and medical masks [40, 41]. The interventions show that N95 respirators gives better protection against viral and bacterial infections, compared to medical masks [40] and that medical masks are better than cloth masks and control [41] (Table 4).

### 3.5 Intervention strategies

#### 3.5.1 Start-ups

Intervention strategies which commonly occurred were that time had been set aside (as a resource) to carry out the interventions in the study [27–31, 33, 34, 36, 37, 42–46, 48, 49, 51, 52] and that the interventions had a clear work structure [27–31, 33, 35–52]. The clear work structure included a clear choice of evaluation method, interventions, and a specific time frame between baseline measurement and follow-up.

Intervention strategies which rarely occurred were employee participation (in planning and designing the intervention) [36, 37, 44, 45, 50] and employee pressure for change (dislike of the current work situation) [44] in the intervention start-up. Studies with a continuous work process (mapping and measures in iterations) were also uncommon [30, 35, 43, 48, 51].

#### 3.5.2 Evaluation methods

The evaluation methods were based on subjective data [27, 28, 30, 31, 33–39, 41–45, 47–52]. Subjective data consisted of self-estimation in questionnaires. Questionnaires used evaluated different aspects of work-related problems. Pain was evaluated with the Nordic Musculoskeletal Questionnaire [27, 31]. Job satisfaction was evaluated with the Minnesota Job Satisfaction Questionnaire [27], Physician Job Satisfaction Scale [51], and the Nurses Job Satisfaction Questionnaire [28]. Psychosocial work factors were evaluated with the Job Content Questionnaire [48], the Karasek’s Job Content Questionnaire [30, 33, 49], the Copenhagen Psychosocial Questionnaire [31, 33], the Effort-Reward Imbalance Questionnaire [48], the DISQ questionnaire [44], and the K6 scale [36]. Exhaustion was evaluated with the Oldenburg Burnout Inventory [34], Copenhagen Burnout Inventory [30] and the Maslach Burnout Inventory [51]. Health was evaluated with the SF-36 [34], Psychiatric Symptom Index [30], Nottingham Health Profile [30], Center for Epidemiologic Studies Depression Scale [48], and the Nordic Occupational Skin Questionnaire [49]. Work engagement was evaluated with the Utrecht Work Engagement Scale [34] and work empowerment was evaluated with the Empowerment at Work Scale [51]. A majority of the included questionnaires were evaluated regarding psychometric properties, mainly internal consistency (Cronbach’s alpha).

Objective data included reported injury rates [29, 46, 52], reported needle stick injuries [32, 42, 50], laboratory confirmed infections [40, 41], number of completed checklists [34], contaminated surfaces [35], images of working postures [39, 47] and the use of patient handling devices measured by accelerometers/push buttons [37].

#### 3.5.3 Intervention characteristics

The interventions were considered evidence-based [27, 29–31, 33, 34, 37, 39–41, 44–52] with a multifactorial approach (several interventions at the same time) [27, 29–35, 37, 38, 43–46, 48–52]. Participative interventions were used in twelve of the twenty-six included studies [27, 30, 31, 34, 35, 37, 43–45, 48, 49, 51]. The studies showed positive outcomes to a very high degree, regardless of using subjective or objective data (Table 5).

**Table 5:** Intervention strategies in the individual studies (Y = yes, N = no, U = unknown, NA = not applicable)

Author (year) Items	Abdollahi (2020)	Babaeipour-Divshali (2016)	Black (2011)	Bourbonnais (2011)	Chanchai (2016)	dÉttorre (2016)	El Khamali (2018)	Gordon (2018)	Graeve (2017)	Guay (2016)	Jacobsen (2019)	Jia (2020)	Lee (2013)
**1. Start-up**
Managers –engagement/participation	N/U	Y	Y	Y	N/U	N/U	N/U	Y	Y	N/U	Y	N/U	N/U
Employees –pressure for change	N/U	N/U	N/U	N/U	N/U	N/U	N/U	N/U	N/U	N/U	N/U	N/U	N/U
Employees –participation	N/U	N/U	NA	N/U	N/U	N/U	N/U	N/U	N/U	Y	Y	N/U	N/U
Resources –time	Y	Y	Y	Y	Y	N/U	Y	Y	N/U	Y	Y	N/U	N/U
Resources –knowledge	Y	N/U	N/U	Y	Y	N/U	Y	Y	N/U	Y	Y	N/U	N/U
Resources –equipment	N/U	N/U	Y	N/U	N/U	Y	N/U	N/U	Y	N/U	Y	Y	N/U
Communication –vertical network	N/U	N/U	N/U	Y	N/U	N/U	N/U	N/U	Y	N/U	Y	N/U	N/U
Communication –horizontal network	N/U	N/U	N/U	Y	Y	N/U	N/U	N/U	Y	N/U	Y	N/U	N/U
Clear work structure	Y	Y	Y	Y	Y	N/U	Y	N/U	Y	Y	Y	Y	Y
Continuous work process	N/U	N/U	N/U	Y	N/U	N/U	N/U	N/U	Y	N/U	N/U	N/U	N/U
**2. Evaluation method**
Subjective data	Y	Y	N/U	Y	Y	N/U	Y	Y	Y	Y	Y	Y	Y
Validity and reliability	Y	Y	NA	Y	Y	NA	Y	Y	Y	Y	N/U	Y	N/U
Objective data	N/U	N/U	Y	N/U	N/U	Y	N/U	Y	Y	N/U	Y	N/U	Y
Validity and reliability	NA	NA	N/U	NA	NA	N/U	NA	N/U	Y	NA	Y	NA	N/U
**3. Interventions**
Evidence based interventions (EBM)	Y	N/U	Y	Y	Y	N/U	Y	Y	N/U	N/U	Y	N/U	Y
Tailored EBM	Y	N/U	N/Y	Y	Y	N/U	Y	Y	Y	N/U	Y	N/U	N/U
Time-benefit interventions	N/U	N/U	N/U	N/U	N/U	N/U	N/U	N/U	N/U	N/U	N/U	N/U	N/U
Dynamic/flexible interventions	N/U	N/U	N/U	N/U	N/U	N/U	N/U	N/U	N/U	N/U	N/U	N/U	N/U
Single intervention	N/U	Y	N/U	N/U	N/U	N/U	N/U	N/U	N/U	Y	N/U	N/U	Y
Multifactorial interventions	Y	N/U	Y	Y	Y	Y	Y	Y	Y	N/U	Y	Y	N/U
Participative work process	Y	N/U	N/U	Y	Y	N/U	N/U	Y	Y	N/U	Y	N/U	N/U
**4. Outcome**
Improvement –Subjective data	Y	Y	NA	Y	Y	NA	Y	Y	Y	Y	Y	Y	Y
Improvement –Objective data	NA	NA	Y	NA	NA	Y	NA	Y	Y	NA	Y	NA	Y
Learning process in the workplace	N/U	N/U	N/U	N/U	N/U	N/U	N/U	Y	Y	N/U	N/U	N/U	Y
**1. Start-up**
Managers –engagement/participation	N/U	N/U	Y	Y	Y	Y	N/U	N/U	Y	N/U	N/U	N/U	N/U
Employees –pressure for change	N/U	N/U	N/U	N/U	Y	N/U	N/U	N/U	N/U	N/U	N/U	N/U	N/U
Employees –participation	N/U	N/U	N/U	N/U	Y	Y	N/U	N/U	N/U	N/U	Y	N/U	N/U
Resources –time	N/U	N/U	Y	Y	Y	Y	Y	N/U	Y	Y	N/U	Y	Y
Resources –knowledge	N/U	N/U	Y	Y	N/U	Y	N/U	N/U	Y	N/U	N/U	Y	Y
Resources –equipment	Y	Y	N/U	N/U	N/U	N/U	Y	N/U	N/U	N/U	Y	N/U	Y
Communication –vertical network	N/U	N/U	N/U	Y	Y	Y	N/U	N/U	Y	N/U	N/U	N/U	N/U
Communication –horizontal network	N/U	N/U	N/U	Y	Y	Y	N/U	N/U	Y	N/U	N/U	Y	Y
Clear work structure	Y	Y	Y	Y	Y	Y	Y	Y	Y	Y	Y	Y	Y
Continuous work process	N/U	N/U	N/U	Y	N/U	N/U	N/U	N/U	Y	N/U	N/U	Y	N/U
**2. Evaluation method**
Subjective data	N/U	Y	Y	Y	Y	Y	N/U	Y	Y	Y	Y	Y	Y
Validity and reliability	NA	N/U	N/U	N/U	Y	Y	NA	N/U	Y	N/U	Y	Y	N/U
Objective data	Y	Y	Y	N/U	N/U	N/U	Y	Y	N/U	N/U	Y	N/U	Y
Validity and reliability	Y	Y	N/U	NA	NA	NA	N/U	N/U	NA	NA	N/U	NA	N/U
**3. Interventions**
Evidence based interventions (EBM)	Y	Y	N/U	N/U	Y	Y	Y	Y	Y	Y	Y	Y	Y
Tailored EBM	N/U	N/U	N/U	Y	Y	Y	N/U	N/U	Y	N/U	N/U	N/U	N/U
Time-benefit interventions	N/U	N/U	N/U	N/U	N/U	N/U	N/U	N/U	N/U	N/U	N/U	N/U	N/U
Dynamic/flexible interventions	N/U	N/U	N/U	Y	N/U	Y	N/U	N/U	N/U	N/U	N/U	N/U	N/U
Single intervention	Y	Y	Y	N/U	N/U	N/U	N/U	Y	N/U	N/U	N/U	N/U	N/U
Multifactorial interventions	N/U	N/U	N/U	Y	Y	Y	Y	N/U	Y	Y	Y	Y	Y
Participative work process	N/U	N/U	N/U	Y	Y	Y	N/U	N/U	Y	Y	N/U	Y	N/U
**4. Outcome**
Improvement –Subjective data	NA	N/U	Y	Y	Y	Y	NA	Y	Y	N/U	Y	Y	Y
Improvement –Objective data	Y	N/U	Y	NA	NA	NA	Y	Y	NA	NA	N/U	NA	Y
Learning process in the workplace	N/U	Y	N/U	Y	Y	Y	N/U	N/U	Y	N/U	N/U	Y	N/U

Interventions based on time-benefit analysis (no study), dynamic/flexible interventions (interventions changing during the study, tailoring interventions to current need) [43, 45], and single interventions (as opposed to multifactorial interventions) [28, 36, 39–42, 47] were rare (Table 5).

## 4 Discussion

### 4.1 Result discussion

The results of this systematic literature review support the use of workplace interventions, with regards to planning, organizing and designing the workplace context, to improve work conditions, health, and well-being in hospital settings.

Previous research has shown workplace interventions reduce stress, distress and burnout in physicians and nurses in hospital settings [12–16]. The result of this systematic literature review adds new and broader knowledge with regards to outcomes from workplace interventions in hospital settings. The results support improvements in physical outcomes like pain, discomfort, musculoskeletal disorders and injuries. The results also support improvements in other psychosocial outcomes like work demands, support and job satisfaction. There were also improvements in other outcomes like needle stick injuries, exposure to violence and viral/bacterial infections.

The results are based on intervention studies that were judged as having moderate to good quality overall. The quality of the included RCTs were considered as moderate to good based on their fulfillment of the quality criteria in MMAT (Table 1). The quality assessment of the included RCTs showed a somewhat poorer result for the assessor blinding criteria, with only five of the twelve RCTs clearly stating that the assessors were blinded to the intervention [33, 37, 39, 41, 47] (Table 1). Four of these five RCTs showed positive outcomes [33, 37, 39, 47]. The included non-RCTs were judged as having good quality, based on the high fulfillment of the quality criteria in MMAT (Table 2). The included mixed methods study was judged as having moderate quality, since it only fulfilled three out of five criteria in MMAT (Table 3). The moderate to good quality of the included studies strengthens the value of the results. The studies were conducted in many different countries, with different study designs, different sample sizes, different types of interventions, and different types of outcomes (Table 4). Improvements were shown in almost all the included studies despite a large study heterogeneity, which further strengthens the results supporting workplace interventions as a means to improve work conditions.

The results of this systematic literature review add knowledge with regards to workplace interventions in hospital settings. The workplace interventions were heterogeneous and almost all included different combinations of ergonomic, environmental, psychosocial and organizational interventions. This finding corresponds with van der Beek et al. supporting that considerations must be given to physical, psychological, and psychosocial factors etc. to understand risk exposure [2]. Generalizability of workplace interventions has been questioned in previous research, since interventions like these usually are tailored to a specific organizational context [53]. The results of this systematic literature review support this criticism. The result supports that simple problems can, in some cases, be solved with simple interventions. An example of a simple intervention is changing operating table heights to decrease work discomfort in anesthetists [39, 47]. Most of the included studies contained complex problems that were solved with combinations of interventions. This illustrates the importance of adapting intervention strategies and combinations of interventions to the specific contextual problems at each workplace, supporting a pragmatist complexity approach in workplace interventions.

The results of this study also add knowledge with regards to intervention strategies. In the design of workplace interventions, a clear work structure was created, and time was set aside to enable the intervention to be conducted. The type of problem and level of complexity were addressed when choosing an evaluation method to ensure that relevant perspectives were included in the evaluation. The interventions were based on some form of evidence, had a multifactorial approach (several interventions at the same time) and a participative approach (employer and employee participating in identifying and solving work environment problems).

### 4.2 Strengths and limitations

When interpreting the results of this review, the following aspects should be considered. To ensure the rigor of our study, the literature search strategies were developed and conducted by a research librarian (EN), and how we conducted the various stages of the review were reported in accordance with the PRISMA 2020 Guidelines [19]. The search term “health personnel” was used to include professions other than physicians and nurses, which was a strength in this study. A potential problem with focusing interventions towards one profession is that an intervention that leads to positive outcomes in one profession might lead to negative outcomes in another. Such a conflicting result was shown in the study by Michélsen, et al, 2014 [43]. Their intervention led to decreases in mental strain in physicians and midwives but worsened the social climate among nurses (Table 4). Drop-outs of the included studies were not included in the quality assessment with MMAT, which can be seen as a limitation in the review. The included workplace intervention studies in this systematic review had a complex study structure. The complexity of the included studies was a challenge in the data extraction process. Meta-analyses of the results would have been ideal but was impossible due to a large heterogeneity with regards to intervention characteristics, measurement methods and outcomes. The result is therefore presented with a narrative summary [18], which can be seen as a limitation.

### 4.3 Practical implications

There are practical implications for OHS from this systematic review with regards to workplace interventions. The results support OHS to work in compliance with the ILO definition. In the start-up and design of workplace interventions, basic conditions must be established. A clear work structure should be created, and time should be set aside to enable the intervention to be conducted. The type of problem and level of complexity should be addressed when choosing an evaluation method to ensure that the context specific problems are identified. Simple work environment problems can, in some cases, be solved with simple interventions. Combinations of interventions tailored to the context specific problems should be used to solve complex work environment problems. They should be based on some form of evidence, with a multifactorial and participatory approach and iterative processes.

The results of this study show that if these criteria are met in practical OHS support in workplace interventions, positive outcomes can be expected. With adequate intervention strategies and workplace interventions tailored to the workplace, OHS will be able to support employers and employees complying with the UN-SDG promoting safe and secure working environments in hospital settings.

### 4.4 Future research

Future research should focus on the intervention strategies and evaluating health outcomes in relation to the intervention strategies used in the study. Broad evaluations, multiple professions and the whole department (or several departments) should be used instead of, for example, a single nursing ward in order to capture the complexity. Studies with single outcomes, single professions or single nursing wards should be avoided to minimize the risk of non-evaluated negative outcomes.

It is of great importance that future research should be designed to evaluate interventions in relation to the work environment complexity in hospital settings.

## 5 Conclusion

The results of this systematic review support the use of workplace interventions with focus on planning, organizing and designing the workplace to improve work conditions, health, and well-being in hospital settings. The study adds to previous knowledge in the literature that workplace interventions can contribute to broad positive outcomes of the work environment. Combinations of different interventions tailored to the specific organization were used, which makes outcome generalizations of single interventions hard. Important intervention strategies commonly used in the start-up, evaluation, and intervention of successful workplace interventions, were identified. The results suggest that using a pragmatist complexity approach in workplace interventions can improve outcomes by providing clear intervention strategies and combinations of interventions, tailored to the organization and related to the complexity level of the experienced problems.

With workplace interventions with these characteristics OHS will be able to work in accordance with International Labour Organization definitions. OHS will also be able to support employers and employees complying with the United Nations Sustainable Development Goal to promoting safe and secure working environments in hospital settings.

To gain further knowledge about workplace interventions future research should be designed to handle the work environment complexity in hospital settings.

## Ethical considerations

The study has not been reviewed by the Ethical Review Authority since, according to the Swedish Ethical Review Act, this is not required for this type of study.

## Informed consent

Not applicable.

## Reporting guidelines

This study is presented according to the Preferred Reporting Items for Systematic reviews and Meta-Analyses (PRISMA) 2020 statement.

## Conflict of interest

The authors declare that they have no conflict of interest.
